# Genetic parentage reveals the (un)natural history of Central Valley hatchery steelhead

**DOI:** 10.1111/eva.13681

**Published:** 2024-03-21

**Authors:** Laura C. Goetz, Hayley Nuetzel, David L. J. Vendrami, Anne K. Beulke, Eric C. Anderson, John Carlos Garza, Devon E. Pearse

**Affiliations:** ^1^ Department of Ecology and Evolutionary Biology University of California Santa Cruz California USA; ^2^ Fisheries Ecology Division, Southwest Fisheries Science Center National Marine Fisheries Service Santa Cruz California USA; ^3^ Department of Ocean Sciences University of California Santa Cruz California USA; ^4^ Present address: Columbia River Inter‐Tribal Fish Commission Portland Oregon USA; ^5^ Present address: Department of Animal Behaviour University of Bielefeld Bielefeld Germany

**Keywords:** life history traits, Pacific salmonids, parentage‐based tagging

## Abstract

Populations composed of individuals descended from multiple distinct genetic lineages often feature significant differences in phenotypic frequencies. We considered hatchery production of steelhead, the migratory anadromous form of the salmonid species *Oncorhynchus mykiss,* and investigated how differences among genetic lineages and environmental variation impacted life history traits. We genotyped 23,670 steelhead returning to the four California Central Valley hatcheries over 9 years from 2011 to 2019, confidently assigning parentage to 13,576 individuals to determine age and date of spawning and rates of iteroparity and repeat spawning within each year. We found steelhead from different genetic lineages showed significant differences in adult life history traits despite inhabiting similar environments. Differences between coastal and Central Valley steelhead lineages contributed to significant differences in age at return, timing of spawning, and rates of iteroparity among programs. In addition, adaptive genomic variation associated with life history development in this species varied among hatchery programs and was associated with the age of steelhead spawners only in the coastal lineage population. Environmental variation likely contributed to variations in phenotypic patterns observed over time, as our study period spanned both a marine heatwave and a serious drought in California. Our results highlight evidence of a strong genetic component underlying known phenotypic differences in life history traits between two steelhead lineages.

## 1 INTRODUCTION

Species in environments with high temporal or spatial variation may express multiple phenotypic responses to the dynamic biotic and abiotic cues they receive from their current environment (Sommer, 2020; Yamamichi, 2022). In traits with conditional plasticity, environmental conditions trigger a genetically encoded threshold that produces the more appropriate phenotype (Buoro et al., 2012; Phillis et al., 2016; Sommer, 2020). How genetic variation and environmental cues are incorporated into phenotypic expression remains difficult to disentangle despite advances in molecular technology (Sommer, 2020). In species with populations that have been transplanted by humans, how dynamic traits respond depends on the relative response of genetic variation developed in the previous environment to new environmental cues (Yamamichi, 2022).

Steelhead, the migratory anadromous form of the salmonid species *Oncorhynchus mykiss*, exhibits variation in numerous life history traits. Steelhead undergo complex phenotypic, behavioral, and physiological modifications enabling migration from their natal streams to the ocean, where they mature for at least 1 year before returning to their natal streams to spawn. Unlike most other anadromous salmonid species that die following their first reproduction (Christie et al., 2018), *O. mykiss* individuals may survive through multiple reproductive events, and the frequency of iteroparity varies among populations. Populations often contain multiple life history strategies in dynamic proportions, with alternative ecotypes frequently interbreeding (Kendall et al., 2015; Ohms et al., 2013; Olsen et al., 2006; Satterthwaite et al., 2009; Sloat & Reeves, 2014). Both evolutionary and ecological mechanisms influence the life history expression of *O. mykiss* (Kendall et al., 2015; Pearse et al., 2019; Phillis et al., 2016), and important adaptive genomic variation has been identified for key migratory life history traits (Hess et al., 2016; Waples et al., 2022; Waters et al., 2021; Willis et al., 2020). In particular, one genetic region, a 55‐Mb double‐inversion on chromosome Omy05, features ancestral (A) and rearranged (R) variations that have been repeatedly associated with multiple traits, including egg and early juvenile development (Miller et al., 2012; Nichols et al., 2008; Sundin et al., 2005), juvenile growth (Rundio et al., 2021), age at spawning (Beulke et al., 2023), and sex‐specific resident and anadromous migratory strategies (Arostegui et al., 2019; Pearse et al., 2014, 2019). Because of this association with multiple life history traits and population specific differences, the Omy05 inversion complex (hereafter “Omy05”), appears to influence the fast‐slow development continuum, consistent with the important role of “pace of life” in animal life history development, age‐specific mortality, and reproduction (Healy et al., 2019). Thus, comparisons of different genetic lineages inhabiting the same environment can help to elucidate the relative effects of adaptive genetic variation and environmental factors on the expression of life history traits.

California is composed of multiple microhabitats with distinct environmental conditions and comprises the southern extent of the native range of *O. mykiss* (Abadía‐Cardoso et al., 2016; Satterthwaite et al., 2010; Sogard et al., 2012). The construction of dams, which may form impassable barriers to spawning habitat and modify natural streamflows, has contributed to the decline of anadromous *O. mykiss* and other native fishes (He & Marcinkevage, 2017; Lindley et al., 2006). This decline prompted the creation and maintenance of hatchery populations to support anadromous fish in their native ranges. Anadromous salmonid hatcheries most often operate as semi‐captive populations; juveniles are reared on‐site and released to migrate and mature in the ocean before returning as adults that are manually spawned at the hatchery. In the California Central Valley (CCV), four hatchery programs rear and release steelhead in highly regulated watersheds below dams. Notably, one of them was founded with non‐native steelhead of coastal California origin, which are known to be genetically distinct from CCV steelhead (Pearse & Garza, 2015). While regulation of CCV streams by dams homogenizes stream flows throughout the year (Sogard et al., 2012), the CCV displays higher temporal and spatial variation in stream flow and temperature, lower rainfall, higher summer temperatures, and high variation among watersheds (Satterthwaite et al., 2009; Sogard et al., 2012) as compared to coastal California habitats. State‐dependent models predict different *O. mykiss* life history compositions among CCV rivers based on this high environmental variability (Satterthwaite et al., 2009; Sogard et al., 2012). Thus, the CCV study system provides an excellent opportunity to compare variation in life history traits among different genetic lineages now exposed to similar environmental conditions. Furthermore, California experienced an intense drought between 2012 and 2016 (Eschenroeder et al., 2022; Herbold et al., 2018), as well as a marine heatwave in 2015–2016 which severely impacted the ocean conditions experienced by salmonids (Di Lorenzo & Mantua, 2016; Free et al., 2023), providing an additional opportunity to investigate shifts in life history trait patterns in response to a sudden environmental change.

In this study, we investigated life history variation in steelhead from the four hatchery populations in the CCV, three of which were founded from local sources, while the fourth was founded from a distinct coastal steelhead lineage (see “Study System” below). We nonlethally collected fin clips from every spawning steelhead from 2011 to 2019, including during the record‐setting drought and marine heatwave events. These fin clips enabled both population genetic analysis and parentage‐based tagging (PBT; Anderson & Garza, 2006), which has been successfully employed to understand and manage anadromous fish populations (Abadía‐Cardoso et al., 2013; Evans et al., 2018; Horn et al., 2022). We reconstructed pedigrees with 13,576 parent‐offspring trios and 19,043 unique adult steelhead, representing nearly all steelhead spawned at four CCV hatcheries over 9 years, spanning two to three generations. These data allowed us to describe temporal changes in patterns of iteroparity, age at spawning, migration (straying) of hatchery steelhead, and adaptive genetic variation associated with life history over almost a decade, highlighting the interaction between genetic and environmental factors influencing important life history traits.

## 2 METHODS

### 2.1 Study system

**FIGURE 1:**
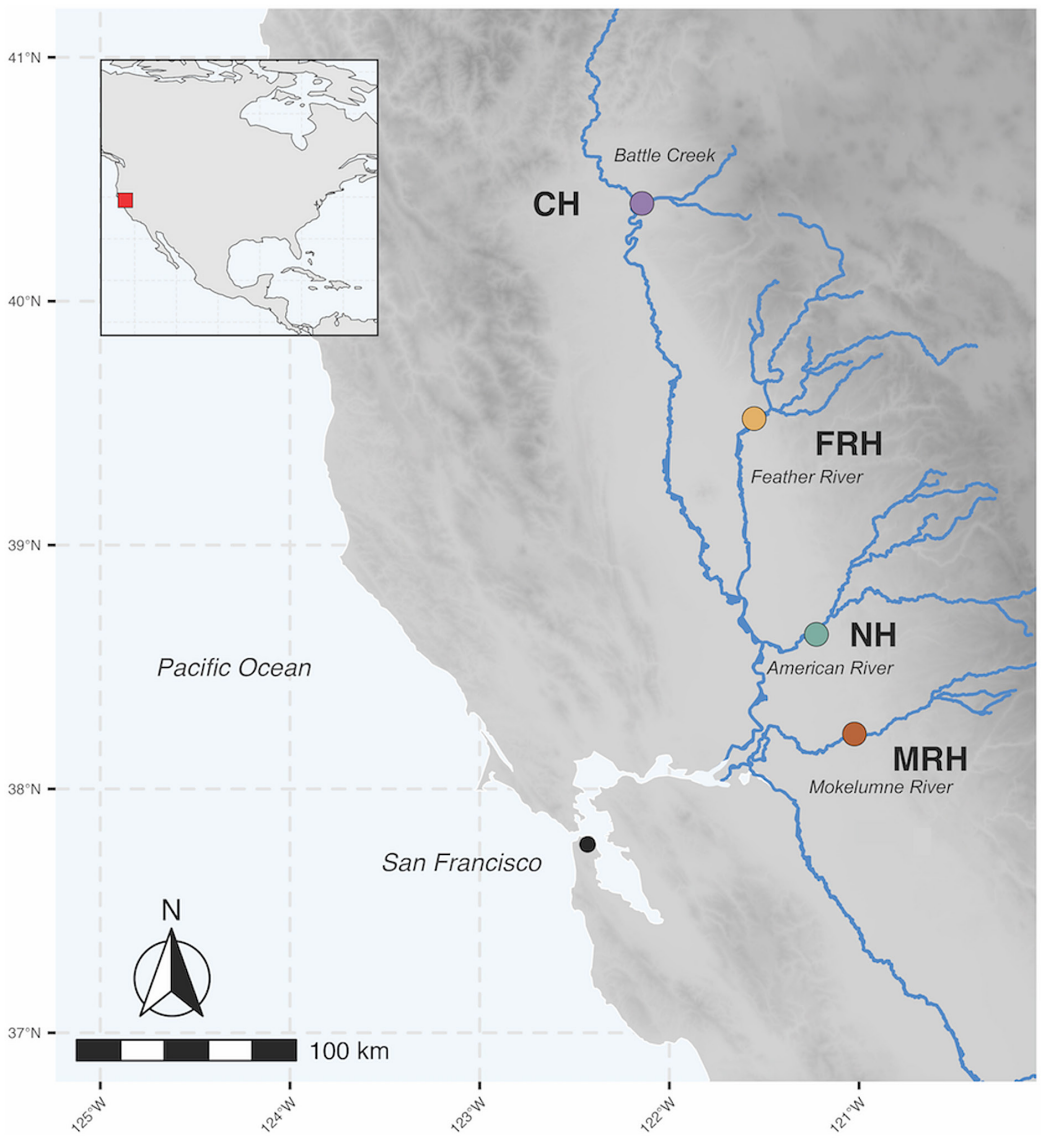
Map of California Central Valley showing locations of hatcheries producing steelhead in relation to San Francisco, CA, USA.

The CCV contains the Sacramento‐San Joaquin River system, a highly impacted region that occupies the central part of California (Figure 1). This low‐elevation area has warmer seasonal temperatures compared with northern *O. mykiss* habitats (Eschenroeder et al., 2022; McEwan, 2001). Landscape and hydrograph alterations from dams built over more than a century have reduced access to over 80% of previous salmonid spawning grounds and homogenized temperature and flow profiles, contributing to decreased numbers of anadromous steelhead (Eschenroeder et al., 2022; He & Marcinkevage, 2017; Lindley et al., 2006). Four hatchery programs produce steelhead in the CCV to mitigate these effects: Coleman National Fish Hatchery (CH), Feather River Hatchery (FRH), Mokelumne River Hatchery (MRH), and Nimbus Hatchery (NH; Figure 1). Situated on different tributaries of the Sacramento River, these four hatcheries capture and spawn returning adult steelhead, incubate the eggs, and rear and release hundreds of thousands of marked (adipose‐fin removed) hatchery‐produced juveniles each year (California HSRG, 2012).

The steelhead spawned at CH, FRH, and MRH were all derived from local CCV populations (“CV‐lineage”) and are part of the California Central Valley Distinct Population Segment (DPS) that is protected as “threatened” under the US Endangered Species Act (NMFS, 2006, 2020). While CH is genetically distinct, FRH and MRH broodstock were previously shown to be almost genetically identical due to increased transfers of FRH eggs from 2002 to 2007, when steelhead returns to MRH were low (Del Real et al., 2012; Pearse & Garza, 2015).

Unlike the other three hatcheries, the NH broodstock was founded by the importation of eggs from coastal California steelhead populations beginning in the 1950s, shortly after the construction of Nimbus Dam (California HSRG, 2012). Consequently, NH steelhead are more genetically similar to coastal steelhead populations than to CV‐lineage hatchery steelhead (Pearse & Garza, 2015). For this reason, steelhead from NH are not included in the CCV steelhead DPS and are managed as a “segregated” program that does not incorporate unmarked (natural‐origin) fish (McEwan, 2001; NMFS, 2006, 2020). However, this does not prevent NH‐origin steelhead from mating in the wild with each other or with listed CV‐lineage steelhead. It is also possible that steelhead migrants from the CCV hatcheries could be spawned at Nimbus, although they are phenotypically distinct and efforts are made to visually identify and exclude them from the broodstock.

Steelhead begin returning to CCV hatcheries in late October and continue through late March. Spawning is typically conducted between December and February, but varies among programs (Figure S1). Not all steelhead that return to a hatchery are spawned. All hatcheries attempt to exclude nonanadromous *O. mykiss* (freshwater resident rainbow trout) by spawning only fish larger than 16 inches (40.64 cm). Rarely, hatchery staff exclude some returning steelhead from spawning because they are phenotypically distinct (a notable case occurred in 2017 at NH when 166 returning fish were not spawned because they were phenotypically dissimilar from NH broodstock, with later genetic analyses confirming these fish were migrants from MRH). At all hatcheries, eggs are stripped from females and fertilized with milt from one or two males. At hatcheries with fewer than 250 returning female steelhead on average per season (MRH and NH), each female's eggs are divided between two males. At the two larger hatcheries (CH and FRH), there are more than 250 returning females on average, each mated with a single male. This practice is intended to mitigate reductions in effective population size (Ne) at the two smaller programs. Hatcheries also differ in how long postspawn steelhead are held before release, which can affect the frequency of repeat spawning within a single season (Fisher & Julienne, 2023). At all four hatcheries, juvenile steelhead are raised on‐site through a year of life before being released either at the hatchery or downstream in the same river; however, size at release varies among the hatcheries. Juvenile steelhead from all four hatcheries are marked by the removal of their adipose fins shortly before release. This practice allows natural steelhead to be visually differentiated from hatchery‐produced steelhead throughout their lifespan, as adipose fins do not regenerate when fully removed.

### 2.2 Sampling and DNA extraction

Tissue samples were taken from each fish spawned at all four hatcheries from 2011 to 2019 and dried on blotting paper in ventilated coin envelopes. The date of spawning, phenotypically identified sex, length, and presence/absence of an adipose fin were recorded for each sample. DNA was extracted from dried fin tissue with QIAGEN DNeasy 96 Tissue Kits following the manufacturer's animal‐tissue protocol using a BioRobot 3000 (QIAGEN Inc.). DNA was then diluted 1:2 in ddH2O prior to genotyping.

### 2.3 SNP loci, genotyping, and basic population genetics analysis

Samples were genotyped with a panel of 96 biallelic SNP markers (Abadía‐Cardoso et al., 2013), including a Y chromosome‐linked marker to determine genetic sex (Brunelli et al., 2008). However, the marker composition of the panel varied slightly over time, with 92 loci genotyped across all years of the study; markers not typed across all years were removed from downstream analyses (Table S1). All individuals were genotyped using TaqMan assays (Applied Biosystems) on 96.96 Dynamic Genotyping Arrays with the EP1 Genotyping System (Fluidigm Corporation) following the manufacturer's protocols. Two negative controls were included in each array, and genotypes were called using SNP GENOTYPING ANALYSIS SOFTWARE V 3.1.1 (Fluidigm).

To evaluate genotyping error rates for each SNP marker, we inferred parent‐offspring trios using parentage analysis (see below) and estimated the minimum genotyping error rate expected to produce the Mendelian incompatibilities observed at each marker across the trios. Of the 23,670 genotyped samples, 83 yielded low‐quality genotypes after the initial round of genotyping (indicated primarily by large fractions of missing genotypes). These samples were re‐genotyped. Any individuals missing more than 10% of loci (fewer than 82 successful genotype calls) were identified and removed.

We utilized the R package “strataG” (version 2.0.2; Archer et al., 2016) to calculate the mean expected and observed heterozygosity averaged over loci for all genotypes in recorded spawned steelhead for each hatchery, excluding individuals that returned to the hatchery but were not spawned. F_ST_ values between years across the study period, both within and between hatcheries, were calculated using strataG on a random subset of 300 broodstock from each hatchery. Next, to evaluate gene flow and population structure among programs, this subset of 1200 individuals (300 from each hatchery) was evaluated in the model‐based clustering program STRUCTURE version 2.2 (Falush et al., 2003; Pritchard et al., 2000) with a hypothesized number of genetic groups of *K* = 2, 3, or 4. Finally, a principal component analysis was conducted on this subset of data to visualize relationships among hatchery programs.

### 2.4 Matching samples, repeat, and iteroparous spawners

We refer to individuals that enter the hatchery and are spawned multiple times within a single year as “repeat spawners.” These can be differentiated from iteroparous individuals that spawn in more than 1 year. Each time any fish is spawned at these hatcheries, a tissue sample is collected and assigned a unique sample ID. Therefore, the same individual may occur multiple times in our dataset with different sample IDs. In order to identify all unique sample IDs belonging to a single repeat spawning or iteroparous individual, we searched our genotype database for samples with identical or near‐identical genotypes using the close_matching_samples() function from the R package “rubias” (Moran & Anderson, 2018). A preliminary analysis with a minimum of 80% of markers with matching genotypes provided a visualization of the distribution of numbers of matching genotypes (Figure S2), from which it was clear that pairs of identical samples shared at least 95% of genotypes. Thus, we identified “clusters” of sample IDs that were from the same individual, including matches observed between different hatcheries, and determined the number of iteroparous and repeat spawners at each program overall, by year, and by sex. To handle cases where more than two sample IDs were from the same fish, we created a graph by defining edges between all pairs of sample IDs that were from the same individual, and then identified all the sample IDs associated with a single fish as members of a connected component in that graph using the R package “igraph” (Csardi & Nepusz, 2006). The significance of spawner sex and hatchery program on the type of multiple spawning event (iteroparity vs. repeat spawners) was determined using Kruskal‐Wallis rank sum tests.

### 2.5 Pedigree reconstruction

To infer the multigenerational pedigree, we conducted parentage analyses, separately for each spawn year and hatchery program included in the study period, using our package *HatcheryPedAgree* (https://github.com/eriqande/HatcheryPedAgree) to implement the SNP Program for Intergenerational Tagging (SNPPIT, Anderson, 2012). SNPPIT assigns each offspring to the most likely parent pair—yielding a parent‐offspring trio—and calculates a false discovery rate (FDR) score for each offspring assignment (to a pair of parents). Before pedigree reconstruction, we removed two markers (genetic sex and Omy_R04944). Based on a preliminary SNPPIT run, the loci Omy_128851‐23 and Omy_131965‐120 displayed an excess of Mendelian incompatibilities (over 2%) and were also removed from subsequent analyses. Final parent–offspring trios were assigned with 92 loci, assuming a genotyping error rate of 0.005 per gene copy (effectively 1% per locus). For pedigree reconstruction, the sample IDs belonging to a single fish were all re‐assigned to be the same as the sample ID associated with the most complete genotype for that fish. In cases where we identified one or more loci scored as different homozygotes among the multiple genotypes in a cluster of genotypes from a single individual, we removed that individual from the data set. To account for potential errors in metadata (i.e. spawn date and/or sex recorded by hatchery staff), we reconstructed pedigrees by using the results of two different SNPPIT runs: one (referred to as the “constrained” run) requiring parents to have the same recorded spawn dates and different recorded sexes, and the second (the “unconstrained” run), in which only spawning year was provided for SNPPIT to determine the possible pairings of parents. Potential parents included fish from all hatcheries, and the list of sample ID clusters was referenced while running SNPPIT to ensure iteroparous and repeat spawners were included in the appropriate potential parent pool based on their multiple spawn dates.

Only trios for which the maximum a posteriori relationship was “parent‐offspring trio” were considered, and only those with FDR ≤0.01 were retained as candidate parent–offspring trios. For most of the offspring, the constrained and unconstrained runs recovered the same parent‐offspring trio assignments. Assignments that differed by SNPPIT run type were largely associated with errors in the metadata (incorrect sex or spawn date). We reconciled these disparities by inspecting the associated metadata and FDR scores of the trios in the two different SNPPIT runs, as described fully in the Results section.

### 2.6 Pedigree‐based analysis

Based on the final pedigrees, parentage assignments and percent of offspring assigned were determined for each parent hatchery, as well as by offspring hatchery, year of spawning, and year of return. The number of offspring per spawning event for females and males was determined, then grouped by type of spawning event (single, iteroparous, and repeat) to calculate both the mean observed reproductive success for each type of spawning event and the mean total observed reproductive success. Repeat and iteroparous spawners that were recorded as different sexes during different spawning events were removed.

Offspring age was calculated for all trios by subtracting the parent spawn year (the year the offspring was born) from the offspring spawn year (the year the offspring returned and spawned). Age distribution was considered by hatchery program and spawn year, as well as by hatchery program and cohort year across years. Including age distribution by hatchery program and cohort year ensures identification of any cohort effects that could influence patterns observed in age distribution by spawn year. Spawn dates were binned into 5‐day units to group spawning steelhead by relative spawn timing. Counts of individuals of each age were noted across recorded lengths (mm) at spawning (size‐at‐age) by program.

Finally, straying rates were calculated for each hatchery program across years. Strays were defined as fish that returned to spawn at a hatchery that differed from that where the parents were sampled.

### 2.7 Omy05

Two SNP loci in our panel (Omy_114448 and Omy_R04944) are located within the inversion complex on chromosome Omy05 (Pearse et al., 2019). Previous analyses have shown that, while Omy_R04944 is nearly perfectly associated with the inversion karyotype, the association of Omy_114448 with the inversion in CCV steelhead is imperfect (Pearse et al., 2014; Pearse & Garza, 2015). Thus, we used locus Omy_R04944 as an indicator of inversion karyotype for all analyses but excluded it from population genetics and pedigree reconstruction. Frequencies of Omy05 karyotypes were determined for each hatchery program to assess patterns related to *O. mykiss* life history in relation to age, sex, and spawn date. Allele frequencies and adherence to the Hardy–Weinberg Equilibrium were evaluated using the R package HardyWeinberg (Graffelman, 2015). Finally, the frequencies of Omy05 genotypes among returning offspring resulting from heterozygous matings (AR × AR) were determined by hatchery and by sex to test for deviations from the expected 1:2:1 genotype frequencies.

## 3 RESULTS

### 3.1 Samples

Fin clips were collected from returning adult steelhead at all four hatcheries from 2011 to 2019, for a total of 23,670 samples (Table 1). The two largest programs, CH and FRH, yielded 9420 and 8218 fin clips, respectively, whereas 2510 samples were collected from MRH and 3522 from NH (Table 1). However, the number of returns at each hatchery varied across years, with all hatcheries experiencing decreases in 2015 and 2016 (Table 1).

**TABLE 1:** Total number of samples received in 2011–2019 from all hatcheries by program and year, as well as the total number of samples removed from genetic analyses due to missing data.

	Program				Total
	CH	FRH	MRH	NH	
Samples genotyped	9275	8013	2493	3467	23,248
Missing loci	145	205	17	55	422
Total sampled	9420	8218	2510	3522	23,670
2011	930	637	207	500	2274
2012	851	756	205	293	2105
2013	891	1512	130	410	2943
2014	878	1499	186	327	2890
2015	1375	580	129	87	2171
2016	452	126	55	503	1136
2017	989	879	647	510	3025
2018	1342	1090	623	399	3454
2019	1567	934	311	438	3250

### 3.2 Data preparation overview

For samples that were re‐genotyped due to low genotyping success, we retained the most complete genotype for those individuals, resulting in 23,670 unique individual genotypes at 92 loci. Setting a minimum of 82 nonmissing loci in the final dataset removed 422 samples (1.78%), leaving 23,248 for further analysis. Recorded phenotypic and genotypic sex were used to determine sex, with nine samples removed for missing both genetic and phenotypic sex.

Identifying samples sharing a minimum of 95% matching genotypes revealed that 4119 fin clip samples could be assigned to 1925 clusters, each representing a single individual that had been sampled between two and seven times due to repeat spawning or iteroparity. The majority of these repeat spawning/iteroparous fish were spawned at FRH (53.74%), while 19.29% were spawned at CH, 16.25% were spawned at MRH, and 10.8% were spawned at NH. In 19 of the 1925 clusters, sex was not consistently recorded for the individual; these individuals were removed. Two samples were identified with mismatching homozygous loci in their cluster of genotypes and removed from further analysis, leaving 23,191 unique individuals for pedigree reconstruction.

### 3.3 Population genetics

**FIGURE 2:**
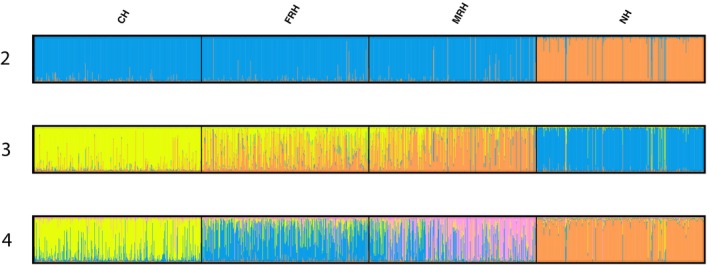
STRUCTURE (Falush et al., 2003; Pritchard et al., 2000) results from 1200 individuals (300 per program) for the hypothesized number of genetic groups *K* = 2, 3, 4.

Estimates of heterozygosity were determined from 22,765 recorded spawned broodstock. Rates of heterozygosity at all programs fluctuated over time, but NH had higher estimated observed and expected heterozygosities than all three CV‐lineage hatcheries in all years (Table S2). F_ST_ and analysis with the program STRUCTURE showed CH, FRH, and MRH are most similar to each other, while NH has the most genetically distinct fish, consistent with their coastal origin (Figure 2; Figures S3 and S4; Table S3). Interannual genetic divergence was lower at CH and FRH, likely due to their larger effective population sizes (Table S3). Notably, MRH and FRH were most similar at the beginning of the study period but became more distinct over time (Figure S4; Table S4).

### 3.4 Iteroparity and repeat spawning

Kruskal‐Wallis rank sum tests revealed that the rate of iteroparity and repeat spawning was significantly different across hatchery programs and between sexes (by hatchery: chi‐square = 52.581, df = 4, *p*‐value = 1.043e‐10; by sex: chi‐square = 27.212, df = 4, *p*‐value = 1.801e‐05). The overall rate of iteroparity was low to moderate (range = 6.3%–14.6%) at all three CV‐lineage hatcheries and was strongly female biased (88.5% of iteroparous spawners were female, Table 2; Tables S5). In contrast, the coastal‐lineage NH had a low proportion of both male and female iteroparous spawners (0.2%; Table 2). Six individuals returned to and spawned at more than one hatchery on different spawn dates. Two of the six individuals were spawned within the same year at different programs: in 2017, one fish spawned at NH and FRH, and in 2018, one fish spawned at CH and FRH. Three individuals were spawned at NH in 2011 and at CH in 2012, and one fish was spawned at MRH in 2018 and NH in 2019.

**TABLE 2:** Counts for single, iteroparous, and repeat spawning for each hatchery program overall, and by sex. Percentages of uses (iteroparous, once, or repeat spawn) for each program and by sex were calculated from the total count of individuals per program, or total females and males.

Use	Sex	CH	FRH	MRH	NH
Iteroparous	Total	588 (6.3%)	667 (8.2%)	364 (14.6%)	7 (0.2%)
	Female	532 (11.1%)	490 (13.4%)	332 (27.6%)	3 (0.2%)
	Male	56 (1.2%)	177 (4%)	32 (2.5%)	4 (0.2%)
Once	Total	8492 (91.4%)	5821 (71.7%)	1829 (73.3%)	3024 (87.3%)
	Female	4146 (86.7%)	2981 (81.5%)	871 (72.4%)	1553 (98.4%)
	Male	4346 (96.5%)	2840 (63.7%)	958 (74%)	1471 (78%)
Repeat	Total	208 (2.2%)	1626 (20%)	304 (12.2%)	433 (12.5%)
	Female	106 (2.2%)	185 (5.1%)	0 (0%)	22 (1.4%)
	Male	102 (2.3%)	1441 (32.3%)	304 (23.5%)	411 (21.8%)

Kruskal‐Wallis	Chi	df	*p*‐Value
Use ~ program	52.581	4	1.04E‐10
Use ~ sex	26.215	4	2.86E‐05

Repeat spawning of the same fish multiple times within a year also varied among hatcheries and across years and was strongly male‐biased (Table 2). FRH had the highest overall rate of repeat spawning (20.0%; Table 2), with a notable reduction from 2016 onward, consistent with changes in management practices (Table S6). The highest rate of repeat spawning within a single season occurred at MRH in 2013 at 50.77% (Table S6). The lowest proportion of repeat spawners was observed at CH (2.24%) in roughly equal numbers of males and females (Table 2).

### 3.5 Pedigree reconstruction

The pedigrees inferred from the unconstrained SNPPIT runs, as well as the runs constrained by sex and spawn date, were each reconstructed from 23,191 unique individuals. The number of trios selected for the final pedigree based on matching spawning metadata and statistical requirements were as follows: 13,657 trios from the constrained run had a maximum a posteriori relationship of “parent‐offspring trio” and an FDR ≤0.01. Over 93% (12,774) of these trios were also found to be statistically supported trio assignments in the unconstrained run, while 883 trios were discrepant between the constrained and unconstrained runs. Of these discrepant trios, 818 offspring did not have statistically supported assigned parents in the constrained run—likely due to errors in the metadata—but were assigned parents that met our confidence criteria in the unconstrained SNPPIT run and were therefore retained. Removing an additional 16 improbable trios with sex or spawning date conflicts left 13,576 trios with confident assignments.

### 3.6 Pedigree‐based analysis

The percentage of spawning steelhead confidently assigned to the pedigree varied by program and year. Table S7 provides details of parentage assignments, total offspring, and number of assignments by cohort and return year.

**FIGURE 3:**
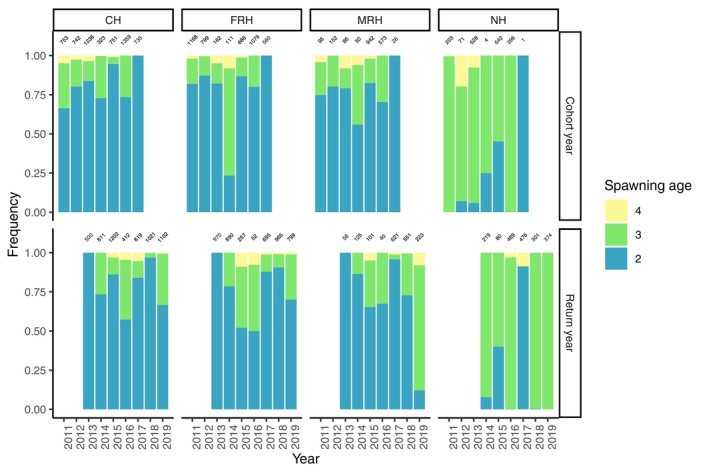
Age structure by program for cohort (above) and return (below) years, with counts of steelhead per year above bars. Note that all fish in return year 2013 and return year 2017 are identified as 2‐year‐olds due to the beginning and ending of the study sampling period for parents in 2011 and offspring in 2019.

Given the filtered parentage assignments, we calculated the age distribution among the spawners at each program. Returning steelhead spawned at two through 6 years of age, with fewer numbers of older fish (Table 3). Age structure varied among programs, with CV‐lineage hatcheries featuring predominantly age‐two steelhead and the coastal‐lineage NH dominated by age‐three steelhead (chi‐squared = 65.25, df = 4, *p*‐value = 2.282e‐13; Figure 3; Table 3). Notably, in 2017, NH had an increased fraction of age‐two spawners return and almost no age‐three spawners (Figure 3). Age structure also varied significantly across programs by sex, with females spawning at older ages than males (chi‐squared = 151.38, df = 5, *p*‐value < 2.2e‐16; Table 3), and by cohort and return year (chi‐squared = 337.47, df = 5, *p*‐value < 2.2e‐16; chi‐squared = 337.64, df = 5, *p*‐value < 2.2e‐16; Figure 3; Table 3).

**TABLE 3:** Counts and percent of steelhead at age at spawning by program and sex; Kruskal‐Wallis results for age‐based results.

Age at spawning	Sex	CH	FRH	MRH	NH
2	Female	2249 (74.8%)	1722 (78.1%)	665 (67.4%)	90 (11.2%)
	Male	2560 (87.9%)	2109 (89.2%)	830 (88.4%)	193 (20.4%)
3	Female	660 (22%)	438 (19.9%)	297 (30.1%)	683 (85.2%)
	Male	322 (11.1%)	243 (10.3%)	100 (10.6%)	724 (76.6%)
4	Female	85 (2.8%)	41 (1.9%)	23 (2.3%)	27 (3.4%)
	Male	23 (0.8%)	12 (0.5%)	9 (1%)	28 (3%)
5	Female	11 (0.4%)	4 (0.2%)	0 (0%)	0 (0%)
	Male	5 (0.2%)	0 (0%)	0 (0%)	0 (0%)
6	Male	1 (0%)	0 (0%)	0 (0%)	0 (0%)
	Female	0 (0%)	0 (0%)	1 (0.1%)	2 (0.2%)

Kruskal‐Wallis	Chi‐squared	df	*p*‐Value
Age ~ program	65.248	4	2.82E‐13
Age ~ sex	151.38	5	<2.2e‐16
Age ~ cohort year	337.47	5	<2.2e‐16
Age ~ return year	337.64	5	<2.2e‐16
Age ~ median spawn date	44.673	10	2.49E‐06

**FIGURE 4:**
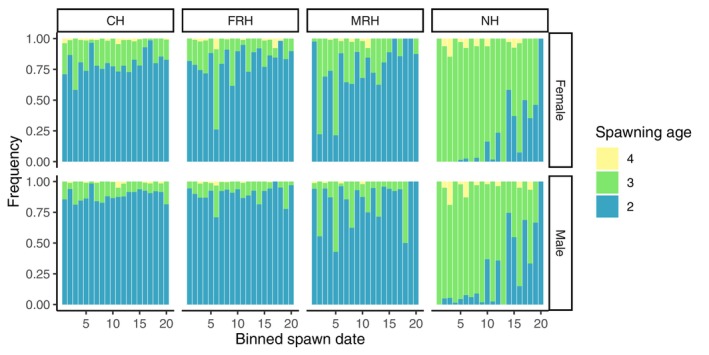
Spawn dates binned over 10‐day intervals and separated by sex over all spawning seasons. Sample sizes for sex and age classes for each binned spawn dates are provided in Table S9.

Comparing age structure across the spawning season with spawn dates grouped into equal, 10‐day, bins revealed striking differences in the spawn dates of age‐two, ‐three, and ‐four spawners at NH, but not at CV‐lineage hatcheries. Mean and median spawn dates by age shift earlier in the season with increasing age of spawner at NH, but not at the CV‐lineage programs (chi‐square = 44.673, df = 10, *p*‐value = 2.491e‐06; Table S8; Table S9). NH showed a clear shift in relative proportion of ages as the spawning season progressed, with older fish returning earlier than younger fish (Figure 4).

Migration among hatcheries was rare but occurred between all programs (Table 4; Table S10). MRH had the highest straying rate due to one significant event, when 165 steelhead that were assigned to parents at MRH in 2015 returned to NH to spawn in 2017. This single event represented 56.66% of all observed straying (Table 4).

**TABLE 4:** Count and frequency of return types in all recorded returning steelhead. Rates of return are provided for both fish returning to their origin program and those that returned to a different hatchery (strays).

	Origin program			
	CH	FRH	MRH	NH
Return program				
CH	5880 (99.4%)	0 (0%)	2 (0.1%)	2 (0.1%)
FRH	11 (0.2%)	4567 (100%)	37 (1.9%)	17 (1%)
MRH	1 (0%)	0 (0%)	1704 (88.5%)	15 (0.9%)
NH	24 (0.4%)	2 (0%)	182 (9.5%)	1713 (98.1%)
Total	5916	4569	1925	1747

### 3.7 Omy05 associations

**FIGURE 5:**
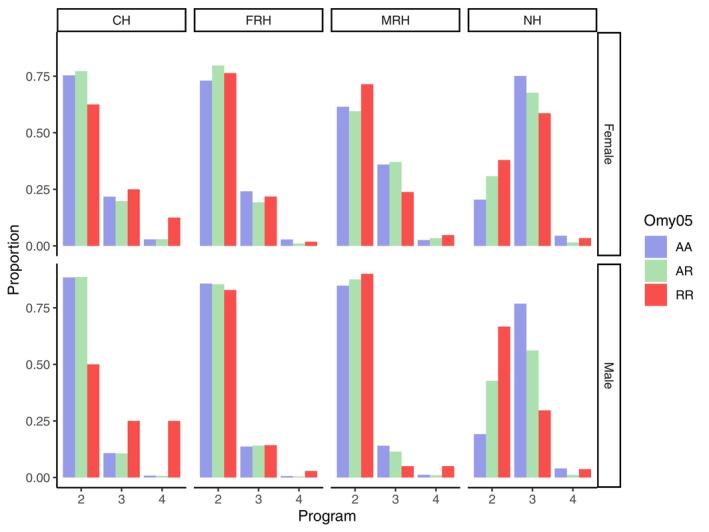
Frequencies of Omy05 genotypes by program, sex, and age at spawning. Sample sizes for each sex, age, and Omy05 genotype class are provided in Table S12.

The marker locus for Omy05 was successfully genotyped for all steelhead sampled from 2015 onward (*N* = 13,090), including 10,293 offspring among the 13,576 inferred trios. Frequencies of Omy05 genotypes were estimated among these individuals overall, by hatchery program, and by hatchery and sex (Table 5). Genotypes AA and AR were most common at all hatcheries, with RR occurring rarely, regardless of sex (Table 5). Using the Haldane Exact test for Hardy–Weinberg equilibrium on the 13,090 genotypes, CH and MRH did not deviate from expected frequencies of Omy05 genotypes, while FRH and NH both had slight, but significant, heterozygote excess (Table 5). We identified 465 returning offspring resulting from AR × AR pairings across all hatcheries, with statistically significant deviations from expected Mendelian Omy05 genotype frequencies overall and for both males and females across all programs, reflecting an excess of heterozygotes and a deficit of RR homozygotes relative to expected Mendelian proportions (Table S11). Age at spawning was not associated with Omy05 genotype among CV‐lineage broodstock, but older coastal‐lineage steelhead from NH were more likely to have AA or AR genotypes, while younger fish had proportionally more RR genotypes (Figure 5; Table S12).

**TABLE 5:** Total counts and frequency of Omy05 genotypes by program and by program and sex. Hardy–Weinberg Equilibrium results are included.

Omy05	Sex	CH	FRH	MRH	NH
AA	Total	5030 (86.6%)	2133 (60.3%)	1259 (70.7%)	1186 (60.4%)
	Female	2558 (86.3%)	1000 (59%)	630 (71.8%)	507 (61.6%)
	Male	2472 (86.8%)	1133 (61.6%)	629 (69.7%)	679 (59.5%)
AR	Total	760 (13.1%)	1272 (36%)	476 (26.7%)	709 (36.1%)
	Female	395 (13.3%)	622 (36.7%)	227 (25.9%)	279 (33.9%)
	Male	365 (12.8%)	650 (35.3%)	249 (27.6%)	430 (37.7%)
RR	Total	20 (0.3%)	130 (3.7%)	45 (2.5%)	70 (3.6%)
	Female	10 (0.3%)	73 (4.3%)	21 (2.4%)	37 (4.5%)
	Male	10 (0.4%)	57 (3.1%)	24 (2.7%)	33 (2.9%)
HWE results	D	7.538726	35.98472	−0.006179775	21.70496
	*p*‐Value	0.1506513	0.000352093	1	0.004105467

## 4 DISCUSSION

We characterized patterns of variation for several important life history traits in the four steelhead hatchery programs present in the CCV from 2011 to 2019, which include two genetically distinct steelhead lineages. Despite inhabiting the CCV environment since the 1950s, we found that the coastal‐origin steelhead at NH maintained genetic and phenotypic distinction from CV‐origin hatchery steelhead, including for key life history traits. Adaptive genetic variation genotyped on chromosome Omy05 varied among populations and showed deviations from expected Mendelian proportions, as well as an association between age at spawning and Omy05 genotype that differed between the CV‐lineage hatcheries and the coastal‐origin steelhead at NH. Finally, we observed strong temporal variation in genetic and phenotypic patterns that coincided with major climatic events over the course of the study.

### 4.1 Distinct genetic lineages

The initiation of NH's hatchery program with coastal‐origin steelhead from the Eel and Mad Rivers created a natural experiment, providing the opportunity to evaluate phenotypic and genetic differences that have been maintained over more than 50 years following transplantation to the CCV (California HSRG, 2012; Pearse & Garza, 2015). F_ST_ and STRUCTURE results support that NH broodstock remain genetically distinct from CV‐lineage steelhead (Pearse & Garza, 2015). These results contribute to evidence of ancestral division between coastal and CV‐lineages and suggest low levels of introgression between NH and the other CCV hatchery programs (Nielsen et al., 2005; Pearse & Campbell, 2018; Pearse & Garza, 2015).

We also found differences among hatchery programs in the distribution of adaptive genetic variation. We investigated the *O. mykiss* chromosome Omy05 chromosomal inversion that has been associated with adaptive growth and developmental traits, as well as migration strategy (Miller et al., 2012; Nichols et al., 2008; Pearse et al., 2014, 2019). NH steelhead appeared distinct from CCV fish in their association of Omy05 genotype with age at spawning, and followed a pattern similar to that observed in coastal steelhead in the Russian River (Beulke et al., 2023). Beulke et al. (2023) found a significant association between age at maturity and Omy05 genotype in males, with the R haplotype more frequent in younger spawners. In NH steelhead, AR and RR genotypes were also proportionally more frequent in age‐two spawners than among older age classes for both sexes.

Within all CCV steelhead hatchery programs, the RR genotype associated with expression of faster development and residency (Pearse et al., 2019; Rundio et al., 2021) was found at low frequencies, consistent with previous observations that southern *O. mykiss* populations below barriers to anadromy possess high frequencies of the A haplotype at Omy05, particularly in the CCV (Abadía‐Cardoso et al., 2019; Eschenroeder et al., 2022; Leitwein et al., 2017; Pearse et al., 2019; Pearse & Campbell, 2018). In addition, Omy05 genotype frequencies significantly deviated from HWE in two programs (FRH and NH). Similarly, across all programs, there was a significant deviation from the expected 1:2:1 Mendelian ratio of Omy05 genotypes in returning offspring from matings between AR parents. Together, these patterns suggest that the phenotypic effects of Omy05 variation lead to genotype‐specific disassortative mating, growth, or survival, independent of genetic lineage, supporting its role in the developmental “pace of life” (Healy et al., 2019). However, the specific mechanisms driving this selection remain unclear. Future work will investigate genomic regions Greb1L/Rock1, vgll3, and six6, which have previously been significantly associated with adult migration timing and age at maturity in salmonids (Ayllon et al., 2015; Thompson et al., 2020; Waples et al., 2022; Waters et al., 2021; Willis et al., 2020, 2021).

Because NH steelhead originate from a coastal lineage and are not included in the Central Valley Steelhead Distinct Population Segment, managing agencies in California prohibit interbreeding of NH broodstock with any returning unmarked or visually apparent CV‐lineage steelhead to prevent the introgression of coastal‐adapted genetic architecture into CV‐lineage gene pools (California HSRG, 2012). Our population genetic analyses confirm introgression between programs occurs rarely, but our pedigree also identified a large straying event of 188 CV‐lineage steelhead (most originating from MRH) to NH in 2017. However, these straying steelhead were visually distinguishable from NH broodstock, so they were genotyped and released without spawning. Conversely, we found low levels of straying from NH to the three CV‐lineage hatchery programs across the study period, although NH broodstock are known to interbreed with wild steelhead in the lower American River (Abadía‐Cardoso et al., 2019). The California Hatchery Scientific Review Group (HSRG) ultimately recommended replacing NH broodstock with steelhead suitable for the American River to decrease risks to natural populations, but it is unclear when this will be initiated (California HSRG, 2012; Fisher & Julienne, 2023; NMFS, 2014).

Genetic variation within CV‐origin hatchery steelhead reflected differences in program management strategies, past movement of eggs between programs, and the accumulation of random genetic changes. Human management of spawning steelhead most strongly influenced repeat spawning, with the highest overall rates occurring in FRH. Our pedigree identified FRH steelhead with high rates of spawning multiple times within 1 year until 2016, after which changes in spawning protocols contributed to consistently low rates of repeat spawning (Table S6). We also found evidence of strong population genetic similarity between FRH and MRH, reflecting previous transport of eggs from FRH to MRH (California HSRG, 2012; Pearse & Garza, 2015). However, we also found that MRH became more differentiated from the CV‐lineage programs over time, suggesting that genetic divergence rapidly accumulated after egg transport stopped in 2007. In contrast, we also observed a decrease in F_ST_ values between CH and FRH. These small changes in population structure over time suggest genetic drift acting in local, differentiated pools with limited interbreeding.

### 4.2 Patterns of phenotypic variation

Our analyses also confirmed striking life history differences between NH and the other hatchery populations. Specifically, compared to CV‐lineage steelhead, NH steelhead consistently spawned at older ages, stratified spawn timing by age, typically spawned only once (semelparity), and possessed significant associations with age at spawning. These traits are all characteristic of coastal steelhead, as shown in hatchery populations in the Russian River, CA (Abadía‐Cardoso et al., 2013; Beulke et al., 2023).

The hatcheries established with CV‐lineage broodstock feature a much greater proportion of age‐two spawners, while NH broodstock spawn predominantly at age‐three. This likely reflects NH steelhead adaptation to coastal environments (Abadía‐Cardoso et al., 2013; Eschenroeder et al., 2022; Pearse & Garza, 2015), and has persisted through more than 50 years of hatchery propagation. In addition, parentage analysis revealed that younger fish arrive later in the season compared to steelhead three years and older at NH, but we did not observe this pattern in the CV‐lineage broodstock. This pattern mirrors previous population analyses conducted in coastal steelhead populations, in which fish that mature as age‐two steelhead tend to spawn late in the season (Abadía‐Cardoso et al., 2013; Beulke et al., 2023). This may reflect the prevalence of alternative life history strategies in CV‐lineage steelhead, including use of freshwater and brackish habitats in the Sacramento‐San Joaquin delta rather than undergoing fully anadromous marine migrations (Abadía‐Cardoso et al., 2019; Leitwein et al., 2017; Olsen et al., 2006; Pearse & Campbell, 2018), and highlights the importance of the portfolio effect in maintaining diverse life histories in salmonid populations (Carlson & Satterthwaite, 2011; Price et al., 2021).

Steelhead exhibit plasticity in the number of lifetime reproductive events, with most individuals dying after first reproduction (semelparous), while some live to reproduce in multiple years (iteroparous), with both life history strategies maintained by fitness tradeoffs involving fecundity and mortality (Christie et al., 2018; Seamons & Quinn, 2010). For this reason, all four CV hatchery programs release steelhead after spawning to provide the opportunity for iteroparity (California HSRG, 2012). In our pedigree analyses, hatchery steelhead spawned either only once, more than once within a season (repeat spawning), or in more than one spawn year (iteroparity). Strikingly, NH steelhead differed significantly from CV‐lineage populations when comparing the average number of observed lifetime spawning events. Lower rates of iteroparity occurred in NH steelhead overall (0.2%), which is consistent with previous estimates of iteroparity in coastal California steelhead populations (Abadía‐Cardoso et al., 2013; Beulke et al., 2023). In contrast, rates of iteroparity were higher in the CCV hatchery program populations, with the highest overall rate occurring at MRH (14.58%) and an even higher rate among MRH females (27.6%). Thus, despite sharing a watershed in the CCV, NH hatchery steelhead possessed low rates of iteroparity, suggesting a strong genetic influence from their coastal lineage that has not been largely altered by current environmental conditions.

### 4.3 Environmental influence

Plasticity in life history traits enables expression of more appropriate phenotypes based on environmental cues. The individual's response depends on the heritability of the conditional response threshold sensitivity, in addition to environmental conditions. The most optimal phenotype best balances producing the largest number of offspring possible and maximizing their probability of surviving to spawning (Satterthwaite et al., 2009, 2010). Considering return timing and age at spawning, this decision depends on growth and successfully surviving emigration. Important environmental cues trigger genetically encoded thresholds that initiate phenotypic expression to optimize survival and reproduction in the local environment (Reid & Acker, 2022; Sogard et al., 2012; Sommer, 2020). Environmental cues, such as the difference between relative streamflows at release and on returning to spawn (release and return stream‐flow differentials), smolt release location, route complexity, and water‐chemistry variation, significantly affect both growth rates and emigration survival, thus influencing steelhead life history selection (Kendall et al., 2015; Satterthwaite et al., 2009, 2010; Sturrock et al., 2019). Our results show that genetic differences in environmental threshold sensitivity may persist in novel environments for many generations.

Temporal variation in CCV watersheds likely influenced the distribution of age at spawning in all four hatchery programs, though identifying specific environmental factors and their interactions with disruptions in genetic and phenotypic patterns remains challenging. The record‐setting 2012–2016 drought in the California Central Valley reduced streamflows by an estimated 85%–90%, with a concordant increase in stream temperatures (Eschenroeder et al., 2022; Herbold et al., 2018). Simultaneously, a strong marine heatwave affected the West Coast of North America in 2014–2016, impacting many anadromous salmonid populations (Di Lorenzo & Mantua, 2016; Free et al., 2023). Sudden relief of the drought in 2017 coincided with higher proportions of age‐two spawners across all programs (Herbold et al., 2018), seen most dramatically in the coastal‐lineage steelhead at NH, where age‐three spawners are typically the most abundant. Furthermore, a combination of NH's proximity to San Francisco Bay and the increased use of downstream smolt‐release sites by all programs during the drought, followed by watershed‐wide flooding in 2017, likely influenced the high proportion of steelhead released from MRH in 2015 to return to spawn at NH in 2017 (Sturrock et al., 2019). These observations highlight the impacts of environmental variability as well as the underlying genetic basis of life history variation. However, because NH is the only hatchery in the CCV that supports coastal‐lineage steelhead, it is unclear exactly how environmental factors differentially impact the expression of life history traits in these divergent lineages.

## 5 CONCLUSIONS

The coexistence of multiple hatchery‐managed steelhead lineages in the CCV provided the opportunity to investigate how different genetic lineages respond to similar environmental cues within a shared landscape. We found that coastal‐lineage NH broodstock have maintained both genetic and phenotypic differentiation compared with steelhead from CV‐lineage programs, despite sharing a watershed for over 50 years. NH steelhead spawned at older ages, maintained lower rates of iteroparity, and showed evidence of novel phenotypic effects of the Omy05 genotype associated with age at spawning. We also observed temporal variation in patterns of life history variation within and among programs, consistent with patterns of climatic variation across years. Collectively, these results highlight the interplay between management practices and biological drivers leading to realized patterns of life history variation in hatchery programs. Our study provides clear evidence that different steelhead genetic lineages may respond differently to novel and changing environments, maintaining strong differences in phenotypic and adaptive genetic variation and life history traits over many generations.

## CONFLICT OF INTEREST STATEMENT

The authors declare no conflict of interest.

## Supporting information


Table S1.
Table S2.
Table S3.
Table S4.
Table S5.
Table S6.
Table S7.
Table S8.
Table S9.
Table S10.
Table S11.
Table S12.

Figure S1.
Figure S2.
Figure S3.
Figure S4.

## Data Availability

Metadata records and genotype data are available on Dryad at doi: 10.5061/dryad.fttdz091g and processed data and analysis tools are available on GitHub at https://github.com/lauracgoetz/CV_steelhead_PBT.
