# Supplementary material for: Genetic parentage reveals the (un)natural history of Central Valley hatchery steelhead
Source: Evol Appl. 2024 Mar 21;17(3):e13681. doi: 10.1111/eva.13681 (PMC10956469; doi:10.1111/eva.13681)
Supplement: Supplementary file 2 — Figure S1. Figure S2. Figure S3. Figure S4. [file EVA-17-e13681-s001.docx]

**Supplementary Figures**


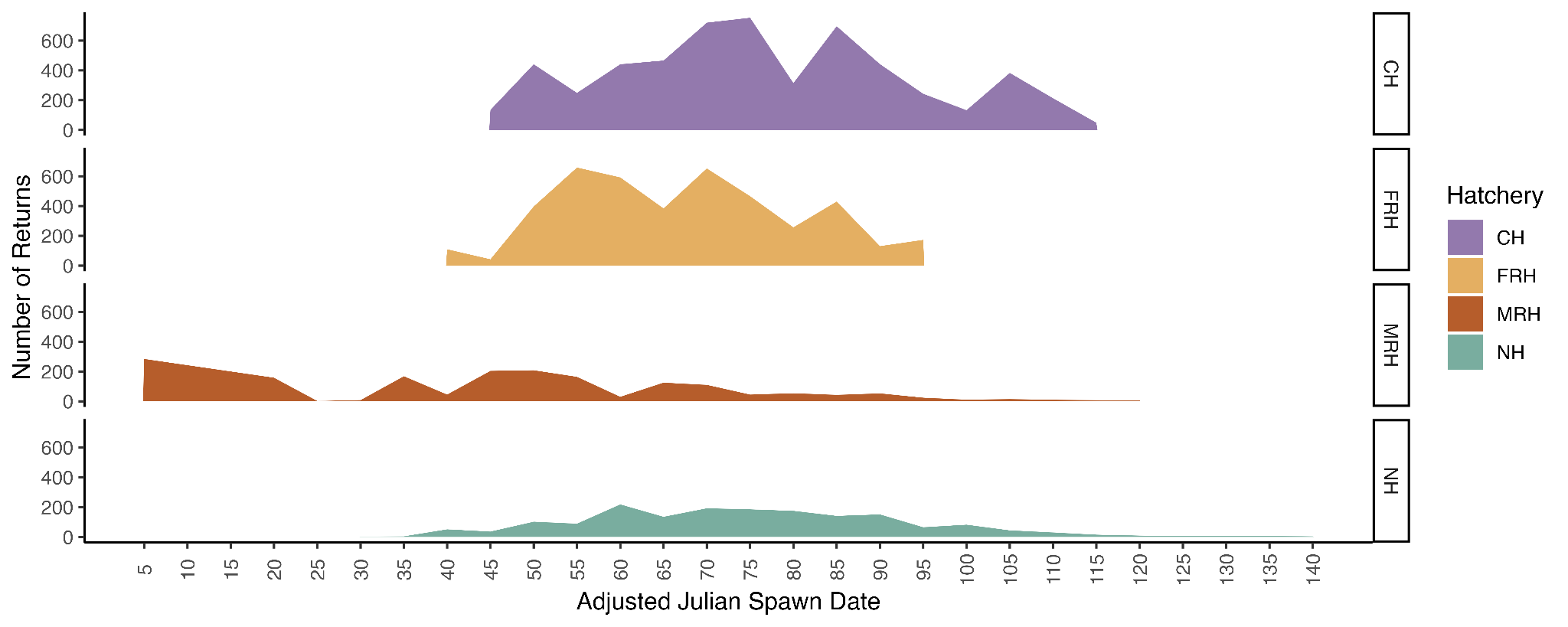


**Figure S1.** Histogram showing the number of spawning steelhead across programs and all spawning seasons. Spawning dates were converted to Julian dates and adjusted to sequential order by spawn season timing, with the x-axis starting at November 1st.


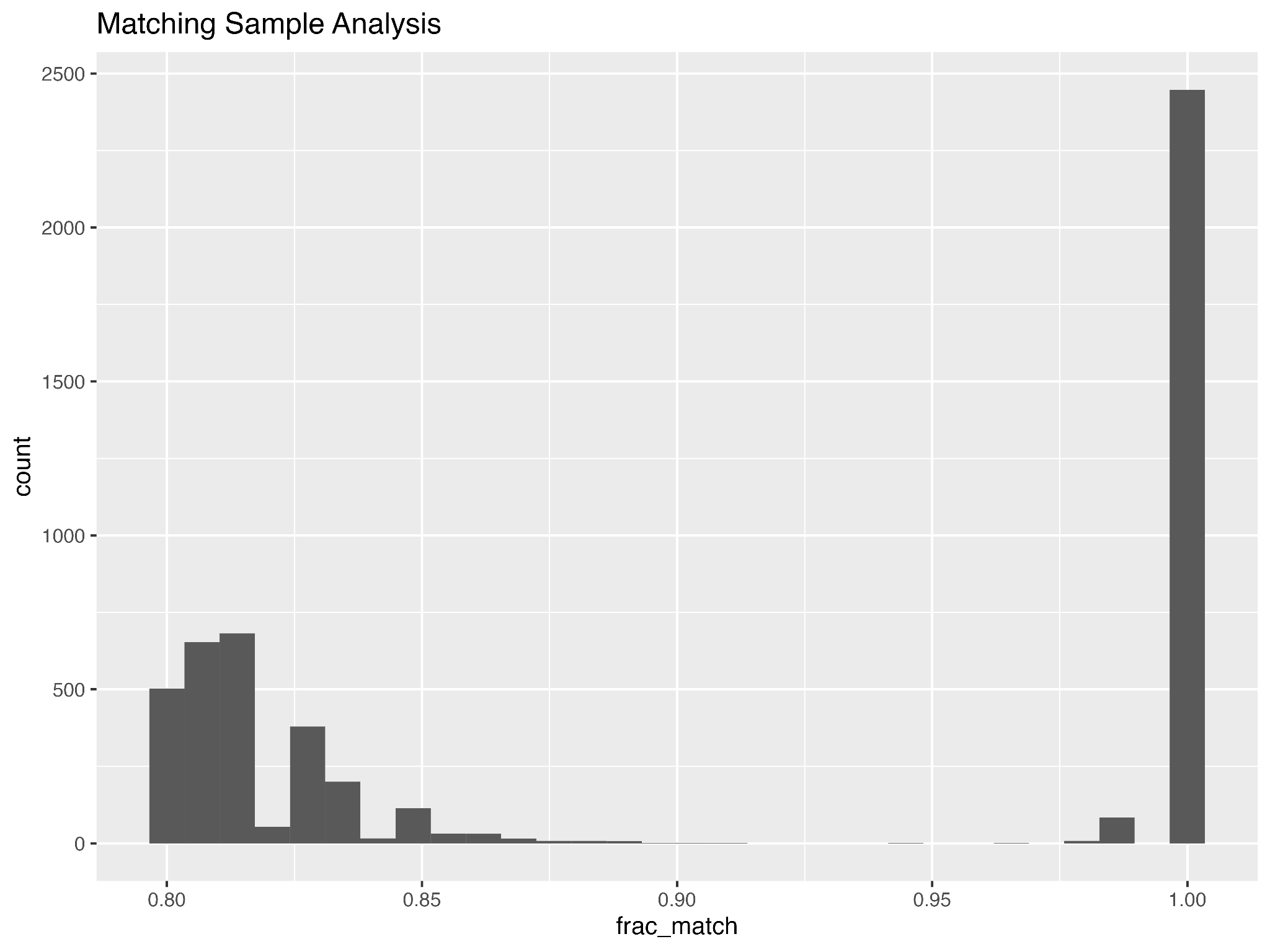


**Figure S2.** Histogram showing the matching sample analysis results using ‘rubias’ (Moran & Anderson 2018) that guided identification of individuals with multiple samples for the parentage analysis. Samples with more than 95% matching genotypes were considered to be from a single individual.

**Figure S3.** PCA results from STRUCTURE across all years, with hatchery programs distinguished by color.
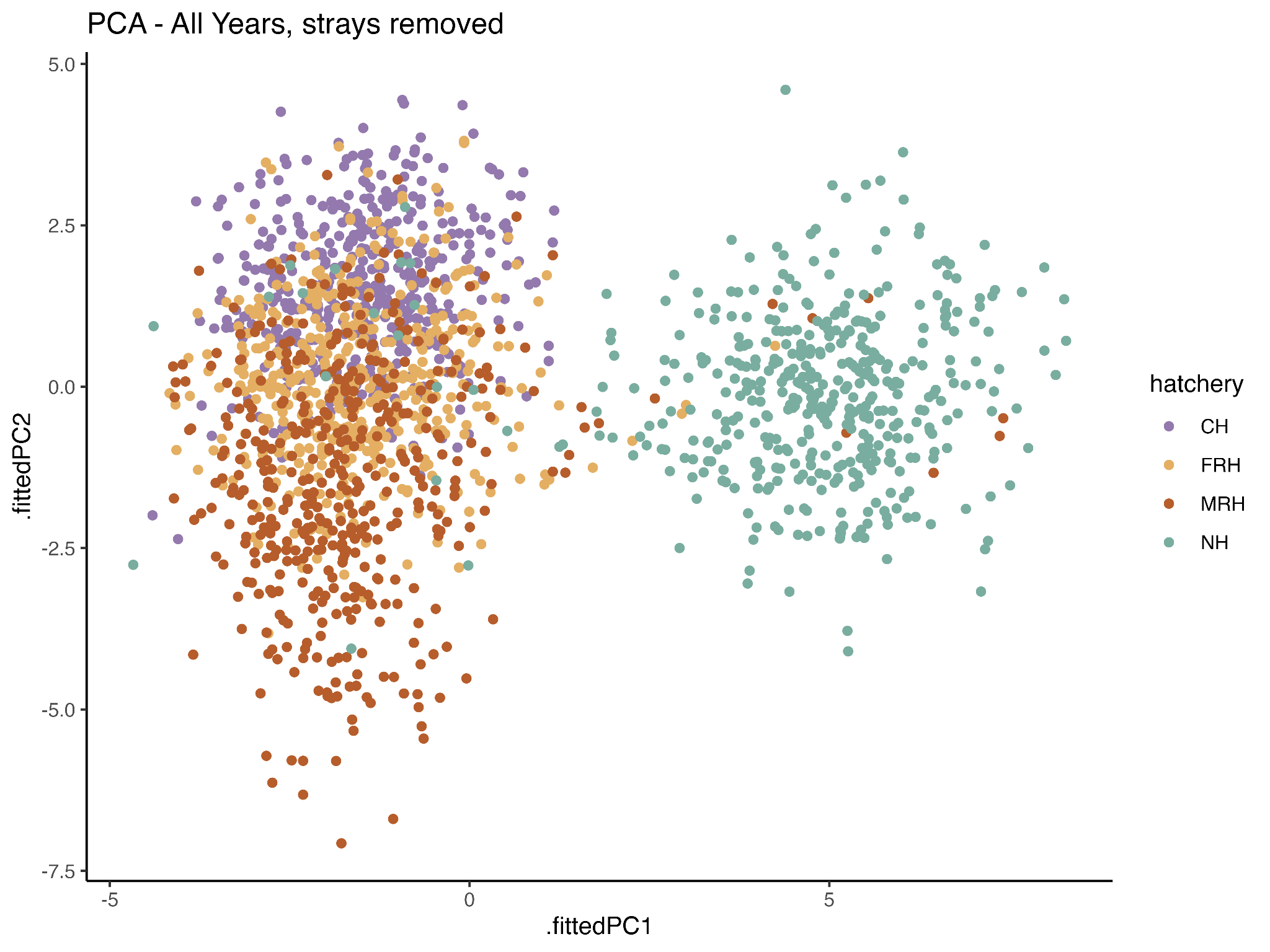


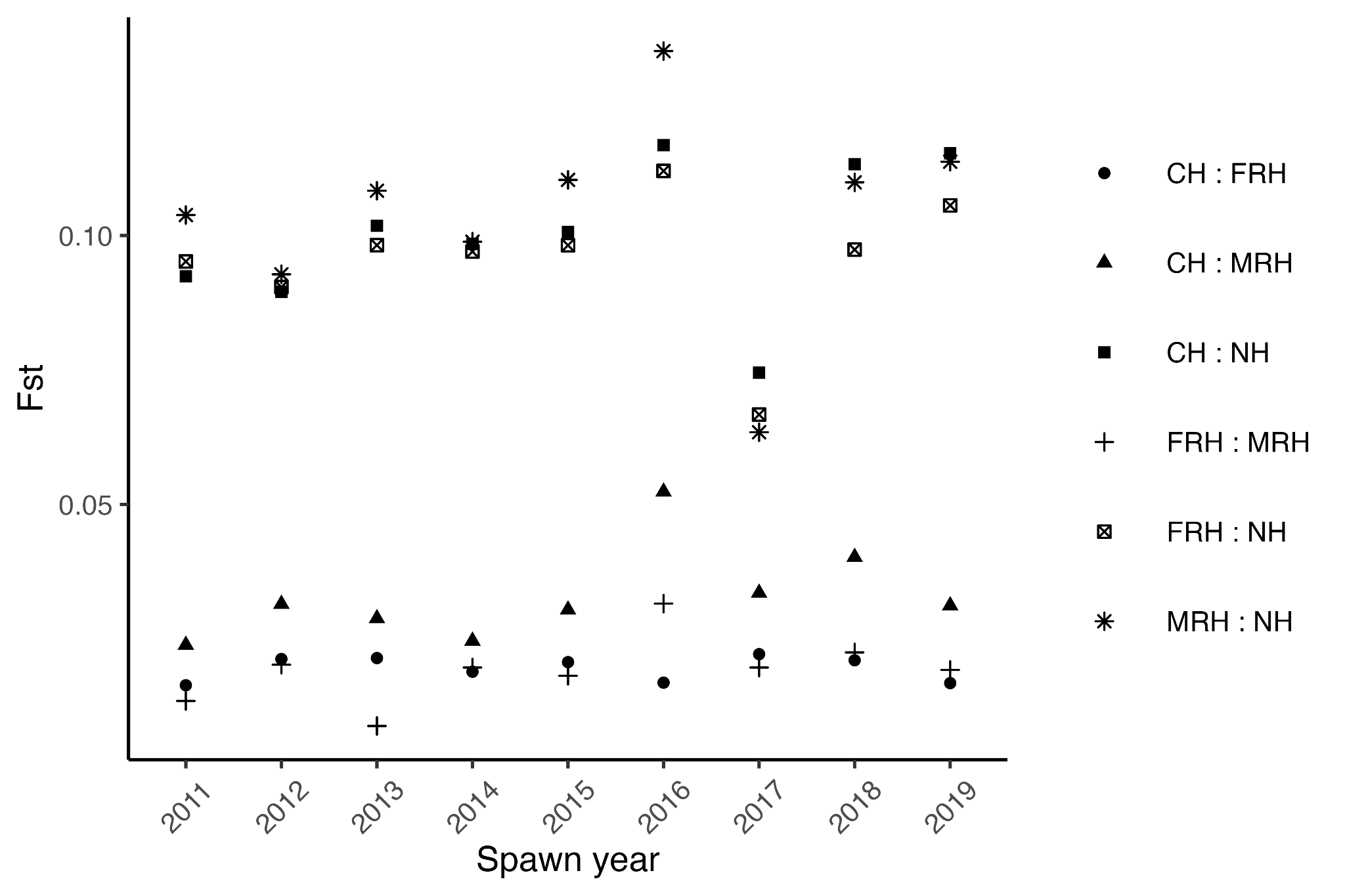


**Figure S4.** Fst between hatchery programs across study period calculated using ‘strataG’ (Archer et al. 2017).
